# Molecular Detection of* Anaplasma* spp. and* Ehrlichia* spp. in Ruminants from Twelve Provinces of China

**DOI:** 10.1155/2016/9183861

**Published:** 2016-12-20

**Authors:** Haixiang Qiu, Patrick John Kelly, Jilei Zhang, Qinghua Luo, Yi Yang, Yongjiang Mao, Zhangping Yang, Jing Li, Hongzhuan Wu, Chengming Wang

**Affiliations:** ^1^Jiangsu Co-Innovation Center for the Prevention and Control of Important Animal Infectious Diseases and Zoonoses, Yangzhou University College of Veterinary Medicine, Yangzhou, Jiangsu 225009, China; ^2^Ross University School of Veterinary Medicine, Basseterre 00265, Saint Kitts and Nevis; ^3^Yangzhou University College of Animal Science and Technology, Yangzhou, Jiangsu 225009, China; ^4^Alabama State University, Montgomery, AL 36104, USA; ^5^Department of Pathobiology, Auburn University College of Veterinary Medicine, Auburn, AL 36849, USA

## Abstract

*Anaplasma* spp. and* Ehrlichia* spp. are tick-transmitted bacteria that are of significant economic importance as they can infect large and small ruminants and also people. There is little information on anaplasmosis and ehrlichiosis in ruminants in China. 16S rRNA FRET-qPCRs were used to screen convenience whole blood samples from 2,240 domestic ruminants in 12 provinces of China for* Anaplasma* spp. and* Ehrlichia* spp. Positive samples were further analyzed with a standard PCR for the* gltA*.* Anaplasma* spp. DNA was detected in the sheep (11.7%; 13/111), goats (81.8%; 219/270), cattle (13.2%; 241/1,830), and water buffaloes (6.9%; 2/29).* Ehrlichia* spp. DNA was detected in sheep (1.8%; 2/111), goats (1.1%; 3/270), and cattle (3.6%; 65/1830) but not in water buffaloes (0/29). Sequencing of* gltA* PCR products showed that* A. marginale*,* A. ovis*,* Ehrlichia canis*, and* Ehrlichia* sp. (JX629807) were present in ruminants from China, while the 16S rRNA FRET-qPCR sequence data indicated that there might also be* A. platys*,* A. phagocytophilum*,* Anaplasma* sp. BL126-13 (KJ410243), and* Anaplasma* sp. JC3-6 (KM227012). Our study shows that domestic ruminants from China are not uncommonly infected with a variety of* Anaplasma* spp. and* Ehrlichia* spp.

## 1. Background

*Anaplasma* spp. and* Ehrlichia *spp. are tick-transmitted, intracellular Gram-negative bacteria that are important animal and human pathogens. The major* Anaplasma *species that impact animal and human health are* Anaplasma marginale*,* A. ovis*,* A. centrale*,* A. bovis*,* A. phagocytophilum*, and* A. platys *[1]. The most important of these in ruminants is* A. marginale* which causes bovine anaplasmosis (formerly gall-sickness) which is associated with fever, anemia, icterus, and often death. The major pathogenic* Ehrlichia* species are* E. canis*,* E. chaffeensis*,* E. ewingii*,* E. muris*, and* E. ruminantium *[2] with the latter causing heartwater in domestic ruminants. This disease is prevalent in Africa, where it causes high mortality (up to 90%) and extensive economic losses [3].

In China, there is little information on the* Anaplasma *and* Ehrlichia *species in domestic ruminants.* Anaplasma bovis, A*.* marginale*, and* A. ovis* have been described in various Provinces, including Xinjiang, Gansu, Henan, Yunnan, Hubei, Guizhou, and Zhejiang [1, 4–7]. In addition,* A. phagocytophilum *has been reported in ruminants (sheep and cattle) in Henan and Xinjiang as well as in dogs and ticks [1, 6–14], and* A. platys* has been detected in red deer and sika deer from Gansu province [4]. In the case of* Ehrlichia *spp. in ruminants in China, an unclassified species has been reported in cattle in Tibet [15] and* E. canis*, a species that infects dogs worldwide, has been reported in sika deer from Gansu [4].

To provide further information on* Anaplasma* spp. and* Ehrlichia* spp. infections in domestic ruminants of China, we carried out a PCR survey for the organisms in blood samples from ruminants in 12 provinces of China. Our findings are reported below.

## 2. Materials and Methods

### 2.1. Blood Samples

This study was reviewed and approved by the Institutional Animal Care and Use Committee of Yangzhou University and animal owners gave written permissions for blood collection.

Between 2007 and 2013, convenience whole blood samples (around 6 mL) from cattle (*n* = 1, 830), water buffaloes (*n* = 29), goats (*n* = 270), and sheep (*n* = 111) were collected in 12 provinces/municipalities of China as described before [16, 17] (Table 1). DNA was extracted from the whole blood samples using a standard phenol-chloroform method as described previously [16] and stored at −80°C until PCRs were performed.

### 2.2. FRET-qPCR

As described previously, FRET-qPCRs for the 16S rRNA of* Anaplasma* spp. [18] and* Ehrlichia* spp. [19], and the mammalian HMBS gene [20] as an endogenous internal control, were performed on a Roche Light-Cycler 480-II PCR Instrument. Positive PCR products were verified by gel electrophoresis and sequenced using forward and antisense primers (BGI, Shanghai, China). Negative controls consisting of sterile molecular grade water were used to detect cross-contamination during DNA extraction and PCR processing.

### 2.3. Standard PCR for the Citrate Synthase Gene (*gltA*)

To further characterize the* Anaplasma *and* Ehrlichia *species detected above, we carried out standard PCRs for the citrate synthase gene (*gltA*) of* Anaplasma* spp. as described previously [21] and for* Ehrlichia* spp. with primers designed for the study (forward primer: GGTTTATGGTGCTTTTCCTAGTGTTGA; reverse primer: TTACAGATTTCTCAGGAGTATATGCCTCC). The PCR products we obtained were verified by gel electrophoresis and sequenced (BGI, Shanghai, China).

### 2.4. DNA Sequence Data Analysis

Compilation and assembly of the multiple sequences generated from each template were performed using the Vector NTI. Sequence alignment was performed with Align (Vector NTI).

## 3. Results

The mammalian HMBS gene endogenous internal control was positive for all samples, indicating that DNA extraction had been successful.

### 3.1. *Anaplasma* spp. DNA in Ruminants

**Figure 1:**
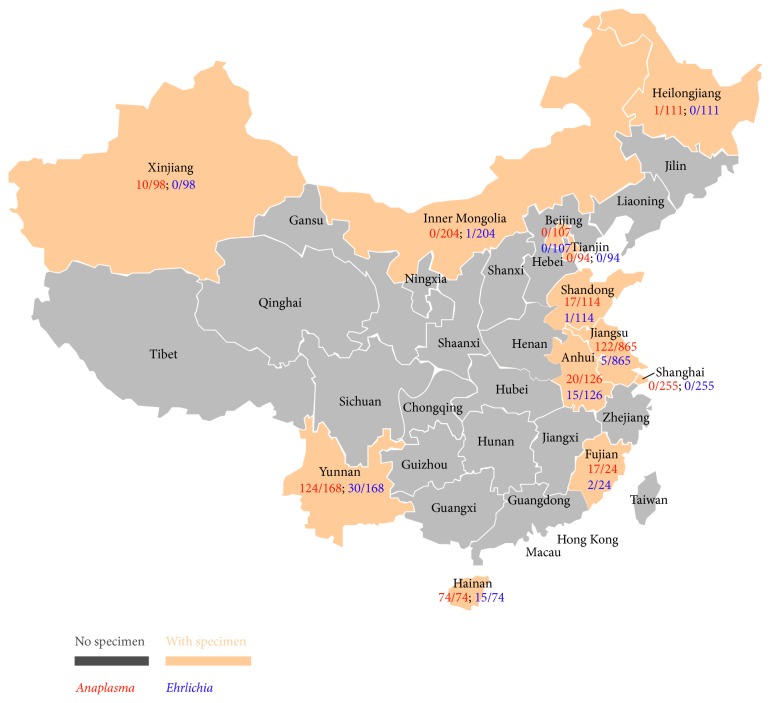
Detection of* Anaplasma* spp. and* Ehrlichia* spp. in ruminants from 12 provinces of China. Blood samples of ruminants (2,240) were collected from twelve provinces (in bisque) of China. The prevalence is shown for* Anaplasma *spp. (red) and* Ehrlichia *spp. (blue).

Overall, 17.2% (385/2240) of the ruminants from 8 of the 12 provinces studied (Table 1 and Figure 1) were positive for the 16S rRNA of* Anaplasma* spp. with copy numbers ranging from 50 to 52,000/mL blood (median 1,720 copies/mL blood). Goats were most frequently positive (81.1%; 219/270), followed by cattle (13.2%; 241/1,830), sheep (11.7%; 13/111), and water buffaloes (6.9%; 2/29).

When we sequenced the positive 16S rRNA FRET-qPCRs, we obtained clean sequencing data for 38 of the samples from cattle (23) and goats (15) (Table 2). The sequences in the cattle were most commonly identical to those of* A. phagocytophilum *(12/23; 52%) and* A. marginale *(10/23; 44%) with positive animals in 4 and 2 of the 9 provinces studied, respectively. Representative sequences were deposited in GenBank for* A. phagocytophilum *(KX279691) and* A. marginale *(KX279690). The one other* Anaplasma *sp. found was in a Wannan cow from Anhui which had a 16S RNA sequence identical to that of a poorly characterized* Anaplasma *sp. BL126-13 (KJ410243). We also found evidence for this organism in three goats from Jiangsu. Other* Anaplasma *spp. we detected in goats were* A. ovis* (KX279688) in Xinjiang (2),* A. platys *(KX279689) in Jiangsu (2), and a poorly characterized* Anaplasma *sp. (KM227012) in Jiangsu (5). The 16S rRNA sequences we deposited in GenBank (KX279683; KX279685) are identical to those of* Anaplasma *sp. (KM227012) and* Anaplasma *BL126-13 (KJ410243), respectively.

**Figure 2:**
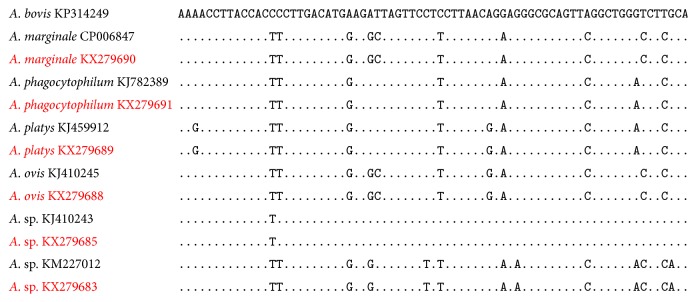
Alignment of the sequences obtained with the 16S rRNA FRET-qPCR we used in our study and those of* Anaplasma* spp. in GenBank. “.” denotes the identical nucleotide sequence to that of* A. bovis*. Organisms with GenBank accession numbers identified in the study are in red.

**Figure 3:**
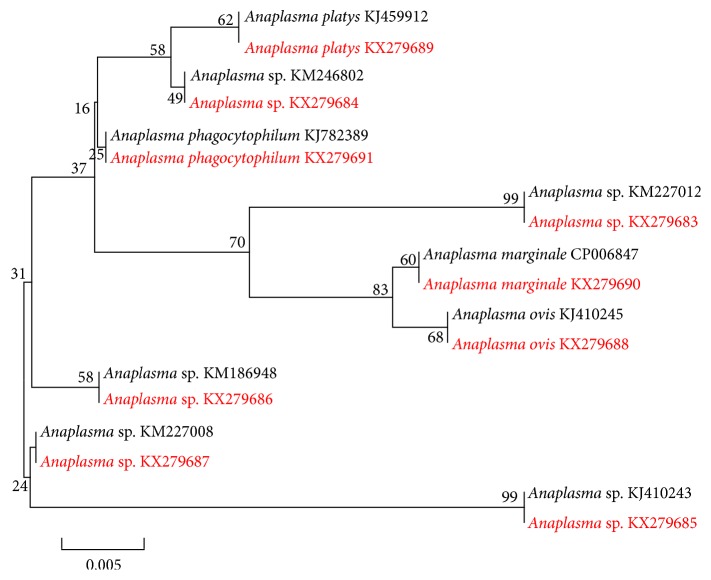
Phylogenetic comparison of* Anaplasma *spp. from ruminants in this study. The 16S rRNA sequences (in red font and accession number) are compared with those of other representing* Anaplasma* spp. (in black font and accession number). Branch lengths are measured in nucleotide substitutions and numbers show branching percentages in bootstrap replicate. Scale bar represents the percent sequence diversity.

Since there is limited polymorphism in the 16S rRNA FRET-qPCR sequences of different ruminant* Anaplasma *spp. (Figure 2), to enable more definitive species differentiation we carried out a PCR and sequencing of the more polymorphic* gltA* on 20% of the 16S rRNA positive samples with the highest copy numbers (range of 610 to 52,000/mL blood; median 2,300). Only two samples provided clean sequencing data with one (16S rRNA FRET-qPCR copy number 52,000/mL blood) from a bovine in Yunnan having 100% identity with* A. marginale* (0/620 mismatches with CP006847.1) and the other (16S rRNA FRET-qPCR copy number 47,700 copies/mL blood) from a goat in Xinjiang having 99.7% identity with* A. ovis* (1/438 mismatches with KJ410284.1). The* gltA* sequence of* A. marginale* we identified was deposited in GenBank under the accession number KX506005 and that of* A. ovis* as KX506006. The 16S rRNA sequences for* Anaplasma *spp. from ruminants in this study are compared with those of other representing* Anaplasma* spp. (Figure 3).

### 3.2. *Ehrlichia* spp. DNA in Ruminants

A total of 70 animals (70/2,240, 3.1%) were positive for DNA of* Ehrlichia* spp. in our 16S rRNA FRET-qPCR with copy numbers varying from 50 to 42,900/mL blood (median 5,100). Cattle were most frequently positive (3.6%, 65/1,830) followed by goats (1.1%, 3/270) and sheep (1.8%, 2/111). None of the water buffaloes (29) were positive. We found* Ehrlichia* sp. positive animals in over half (7/12) the provinces we studied with the highest prevalence in Wuhu (82.4%, 14/17) of Anhui province and lower prevalence in the tropical provinces in the south of China, mainly Hainan (20.3%, 15/74), Yunnan (17.9%, 30/168), and Fujian (8.3%, 2/24). No positive animals were detected from the more northern provinces of Beijing, Shanghai, Heilongjiang, and Tianjin (Table 1; Figure 1).

Standard PCR and sequencing of the* gltA* were performed on samples positive in the FRET-qPCR for 16S rRNA with higher copy numbers, namely, those between 14,400 and 42,900/mL blood (median 16,700). Useable* gltA* sequences were obtained for six of the animals studied (copy numbers 33,100 to 42,900/mL blood, mean 37,600) showing five, all cattle from Yunnan, to be infected with an* Ehrlichia* sp. having an identical sequence (563/563; 100%) to that of a new species closely related to* E. canis* and found in* Rhipicephalus microplus* in the Czech republic (JX629807) [22]. The remaining animal, a goat from Jiangsu, was found to be infected with* E. canis* having a sequence almost identical (549/563; 98%) to that of* E. canis* (Oklahoma strain; AF304143) [23] and a strain found in a dog in Thailand (KJ459920) [24].

**Figure 4:**
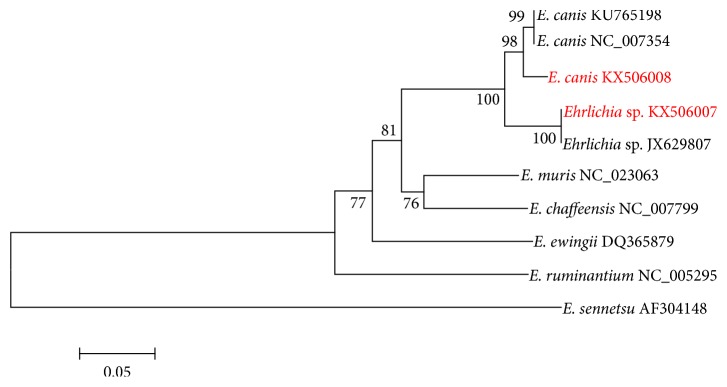
Phylogenetic comparison of* Ehrlichia *spp. from ruminants in this study. The* gltA* sequences (in red font and accession number) are compared with those of other representing* Ehrlichia* spp. (in black font and accession number). Branch lengths are measured in nucleotide substitutions and numbers show branching percentages in bootstrap replicate. Scale bar represents the percent sequence diversity.

The* gltA* sequence of* E. canis* we identified was deposited in GenBank under the accession number KX506008 and that of a representative of the* Ehrlichia* sp. (JX629807) as KX506006. The* gltA* sequences for* Ehrlichia *spp. from ruminants in this study are compared with those of other representing* Ehrlichia* spp. (Figure 4).

## 4. Discussion

Our results are consistent with other PCR studies from China [1, 6–10, 12, 13] showing that domestic ruminants from the country are infected with a range of* Anaplasma *and* Ehrlichia *spp. Differences in the prevalence of animals on the farms we studied were most likely due to differing husbandry practices and tick exposure, with dairy animals and water buffaloes raised intensively having the lowest levels of positivity. Also, although our sample numbers were small, our results indicate that ruminants are generally more commonly infected with* Anaplasma *and* Ehrlichia *spp. in the more southern provinces (Yunnan and Hainan) and along the seaboard (Fujian, Anhui, and Jiangsu) where there are more tropical conditions and tick vectors are expected to be more prevalent. Lower prevalence was found in the cooler northern provinces (Heilongjiang, Beijing, Inner Mongolia, and Xinjiang).

Although we obtained relatively large numbers of animals positive by FRET-qPCR for the 16S rRNA of* Anaplasma *spp., we were only able to amplify a small number of these with the* gltA *primers. We presume this was because of low parasitemia in affected animals and different numbers of target sequences for the PCRs [25], since we could only amplify the* gltA* from animals with high copy numbers in the 16S rRNA FRET-qPCR. It might also, however, have been because of different sensitivities of the PCRs we used as has been described before with molecular detection of different genes in* Anaplasma *spp. [10, 25, 26].

The one* Anaplasma *we definitively identified with the* gltA *PCR was* A. marginale *which is the agent of bovine anaplasmosis, a very common disease of cattle around the world in tropical and subtropical countries [27]. Infections are mainly transmitted by* Rhipicephalus microplus* and although most infections are subclinical, there can be fever and severe anemia resulting in production losses from decreased milk production and abortion. Studies in China have shown that the organism can be found in* Rhipicephalus *(*Boophilus*)* microplus *[28] and also that the organism might be transmitted by* Hyalomma asiaticum *[5]. The organism appears to be widespread in domestic ruminants in China and it has been reported to be a relatively common infection of cattle in southern and northern China [5, 15, 29, 30].

The other* Anaplasma *we definitively identified,* A. ovis*, has also been reported previously in China in goats (15%) from central and southern China [1] and in sheep and goats (41%) in Henan and Xinjiang [6, 7]. This organism is the agent of ovine anaplasmosis which can be transmitted by* Dermacentor nuttalli*,* Hyalomma asiaticum kozlovi*, and* Rhipicephalus pumilio* in China where infections mostly result in subclinical anemia in indigenous animals [31]. Recently, the organism has been shown to infect humans [32] as has a closely related organism in China, putatively named “*Anaplasma capra*” [33].

Although we were not able to use* gltA *sequencing to definitively identify most of the* Anaplasma *spp. we detected with our* 16S rRNA *FRET-qPCR, we could confirm the accuracy of the qPCR in detecting* A. marginale *and* A. ovis*.* Anaplasma marginale *was one of the most common* Anaplasma *species we detected with the 16S rRNA FRET-qPCR along with* A. phagocytophilum *which has also already been described in China where it appears to be common in ruminants [7, 11, 12, 34].* Anaplasma phagocytophilum *is the agent of tick-borne fever of ruminants and is transmitted by* Dermacentor silvarum*,* Haemaphysalis concinna, H. longicornis*, and* Ixodes persulcatus *in China [35, 36]. The organism is now known to infect a wide variety of domestic and wild animals and is the agent of human granulocytic anaplasmosis [27].

Other* Anaplasma *spp. we appear to have identified based on their 16S rRNA sequences include* A. platys*, the agent of infectious canine cyclic thrombocytopenia [3], which has been described in dogs in Asia [24, 37] and in sika deer, goats, and cattle in China [4, 19]. The remaining two* Anaplasma *spp. we appear to have found are as yet only poorly characterized with* Anaplasma *sp. (KM227012) first reported in* Procapra gutturosa, *the Mongolian gazelle, in China and appear to be most closely related to* A. ovis *and* A. centrale *[38].* Anaplasma *sp. BL126-13 (KJ410243) has only been identified in a* Hyalomma asiaticum *from Xinjiang [39]. As we found that these poorly characterized organisms seem to occur relatively commonly, especially in goats, it would seem appropriate that they should be studied further as they could be important pathogens.

A number of* Ehrlichia *spp. have been described in China,* E. chaffeensis*,* E. canis*,* E. platys*,* E. ewingii *(granulocytic ehrlichial agent), and also a novel species closely related to* E. chaffeensis *and* A. marginale* [14, 15, 33, 40]. The most important* Ehrlichia *species infecting ruminants,* E. ruminantium*, is restricted to Africa and some Caribbean islands [19] and has not been reported in China. Of the other* Ehrlichia *known to infect ruminants (summarized in Zhang et al., 2015 [19]), we only found evidence of infection with* E. canis *in the domestic ruminants we studied in China. Although* E. canis* is best known as a very common dog pathogen around the world, infections have also been described in people [41] and in cats [42], and there is thus growing evidence that* E. canis* has a wider host range than previously thought [2, 19, 43]. Our finding of* E. canis *and closely related organisms in a goat and cattle in China further supports this evidence and is consistent with the findings of a study showing that* E. canis* or very closely related organisms are present in domestic ruminants in the Caribbean [19] and also a study showing that* E. canis *occurs in sika deer in China [4]. Further studies are underway in our laboratory to determine the pathogenicity of* E. canis *in domestic ruminants.

In summary, we found DNA of* Anaplasma *spp. and* Ehrlichia *spp. relatively common in the blood of the goats (81.1% and 1.1%, resp.), cattle (13.7% and 3.6%, resp.), sheep (11.7% and 1.8%, resp.), and water buffaloes (6.9% and 0%, resp.) we studied from China. Further, our data from 12 provinces show that a wide range of* Anaplasma *spp. and* Ehrlichia *spp. occur in ruminants in China and further larger scale studies are indicated to determine more accurate prevalence data for these agents and their impact on health and production. The low copy numbers we commonly found indicate that chronic infections are common and this did not enable us to obtain reliable multigene sequence data from most samples. It would appear best, then, for future studies on the presence of* Anaplasma *and* Ehrlichia *spp. to rather be conducted on organisms cultured from infected animals. Ticks should also be considered for such studies as they generally contain relatively high numbers of* Anaplasma *and* Ehrlichia* spp. (2,530 to 970,000/positive tick) [25].

## Figures and Tables

**Table 1:** Molecular detection of *Anaplasma* spp. and *Ehrlichia* spp. in Chinese ruminants.

Animal species	Subspecies/breed		Province	City	*Anaplasma* positivity	*Ehrlichia* positivity
					Positive/total, %	Positive/total, %
Cattle (*n* = 1,830)	*Bos p. taurus*	Holsteins	Anhui	Bengbu	3/109, 2.8%	1/109, 0.9%
		Wannan	Anhui	Wuhu	17/17, 100.0%	14/17, 82.4%
		Holsteins	Beijing	Sanyuan	0/107, 0.0%	0/107, 0.0%
		Holsteins	Jiangsu	Yancheng	1/395, 0.3%	2/395, 0.5%
		Holsteins	Jiangsu	Yangzhou	0/269, 0.0%	1/269, 0.4%
		Holsteins	Heilongjiang	Qiqihar	1/111, 0.9%	0/111, 0.0%
		Simmentals	Inner Mongolia	Chifeng	0/132, 0.0%	0/132, 0.0%
		Luxi	Shandong	Jining	3/42, 7.1%	0/42, 0.0%
		Bohaiblack	Shandong	Binzhou	1/33, 3.0%	0/33, 0.0%
		Holsteins	Shanghai	Shanghai	0/255, 0.0%	0/255, 0.0%
		Holsteins	Tianjin	Tianjin	0/94, 0.0%	0/94, 0.0%

Cattle (*n* = 1,830)	*Bos p. indicus*	Minnan	Fujian	Putian	17/24, 70.8%	2/24, 8.3%
		Leiqiong	Hainan	Haikou	74/74, 100.0%	15/74, 20.3%
		Yunling	Yunnan	Kunming	124/168, 73.8%	30/168, 17.9%

Water buffalo (*n* = 29)	Haizi		Jiangsu	Yancheng	2/29, 6.9%	0/29, 0.0%

Goats (*n* = 270)	Yangtze River Delta White		Jiangsu	Yangzhou	119/172, 69.2%	3/172, 1.7%
	Xinjiang		Xinjiang	Urumqi	10/98, 10.2%	0/98, 0.0%

Sheep (*n* = 111)	Sishui Fur		Inner Mongolia	Xilingol	0/72, 0.0%	1/72, 1.4%
	Wuranke		Shandong	Jining	13/39, 33.3%	1/39, 2.6%

**Table 2:** Source of *Anaplasma* and *Ehrlichia *spp. identified in this study based on 16S rRNA and gltA gene sequences.

Organism	Species	*16S RNA*				Species	*gltA*			
		GenBank #	Numbers	Animal	City, province		GenBank #	Numbers	Animal	City, province
*Anaplasma *spp.	*A. marginale*	KX279690	10	Cattle	Binzhou, Shandong (1)	*A. marginale*	KX506005	1	Cattle	Kunming, Yunnan
					Kunming, Yunnan (9)					
	*A. platys*	KX279689	2	Goat	Yangzhou, Jiangsu					
	*A. phagocytophilum*	KX279691	15	Cattle	Bengbu, Anhui (2)					
					Haikou, Hainan (5)					
					Kunming, Yunnan (4)					
					Wuhu, Anhui (1)					
				Goat	Yangzhou, Jiangsu (3)					
	*A. ovis*	KX279688	2	Goat	Urumqi, Xinjiang (2)	*A. ovis*	KX506006	1	Goat	Urumqi, Xinjiang
	*Anaplasma *sp.	KX279685	4	Cattle	Wuhu, Anhui (1)					
				Goat	Yangzhou, Jiangsu (3)					
	*Anaplasma *sp.	KX279683	5	Goat	Yangzhou, Jiangsu (5)					

*Ehrlichia *spp.	*Ehrlichia *sp.	KX279682	14	Sheep	Inner Mongolia (1)					
				Cattle	Yunnan, Kunming (10)	*Ehrlichia *sp.	KX506007	5	Cattle	Yunnan, Kunming
				Goat	Yangzhou, Jiangsu (3)	*E. canis*	KX506008	1	Goat	Yangzhou, Jiangsu
